# Influence of Al_2_O_3_ Overlayers on Intermolecular Interactions between Metal Oxide Bound Molecules

**DOI:** 10.3390/molecules28124835

**Published:** 2023-06-17

**Authors:** Erica S. Knorr, Cody T. Basquill, Isabella A. Bertini, Ashley Arcidiacono, Drake Beery, Jonathan P. Wheeler, J. S. Raaj Vellore Winfred, Geoffrey F. Strouse, Kenneth Hanson

**Affiliations:** Department of Chemistry and Biochemistry, Florida State University, Tallahassee, FL 32306-4390, USA

**Keywords:** dye sensitized, excimer, ALD, metal oxide, transient absorption

## Abstract

Intermolecular interactions on inorganic substrates can have a critical impact on the electrochemical and photophysical properties of the materials and subsequent performance in hybrid electronics. Critical to the intentional formation or inhibition of these processes is controlling interactions between molecules on a surface. In this report, we investigated the impact of surface loading and atomic-layer-deposited Al_2_O_3_ overlayers on the intermolecular interactions of a ZrO_2_-bound anthracene derivative as probed by the photophysical properties of the interface. While surface loading density had no impact on the absorption spectra of the films, there was an increase in excimer features with surface loading as observed by both emission and transient absorption. The addition of ALD overlayers of Al_2_O_3_ resulted in a decrease in excimer formation, but the emission and transient absorption spectra were still dominated by excimer features. These results suggest that ALD may provide a post-surface loading means of influencing such intermolecular interactions.

## 1. Introduction

Strong intermolecular interactions, especially with large aromatic molecules, can result in emergent multi-molecular phenomena such as aggregation [1], excimer formation [2], triplet–triplet annihilation (TTA) [3], singlet fission [4], and charge transfer [5], among others. These interactions can occur in solution, particularly at high concentrations, but are typically amplified in the solid state when molecules are adsorbed or chemically bound to a surface [6]. Sometimes these interactions are detrimental, as is the case in dye-sensitized devices when bimolecular non-radiative decay pathways or excimer emission outcompetes excited state electron injection [7,8,9]. However, intermolecular interactions can be beneficial when broadened absorption [1], photon upconversion via TTA (TTA-UC) [10,11], and singlet fission [12,13,14] are used to increase solar energy conversion efficiencies. 

Critical to the intentional formation or inhibition of these processes is controlling interactions between molecules on a surface. Historically this has been achieved through changing the solvent [15], metal oxide pre-treatments [16], adding molecular steric bulk [17,18], shifting the position of surface binding groups [19,20], using inert co-adsorbants [21,22,23], or varying the surface loading density [15].

**Figure 1:**
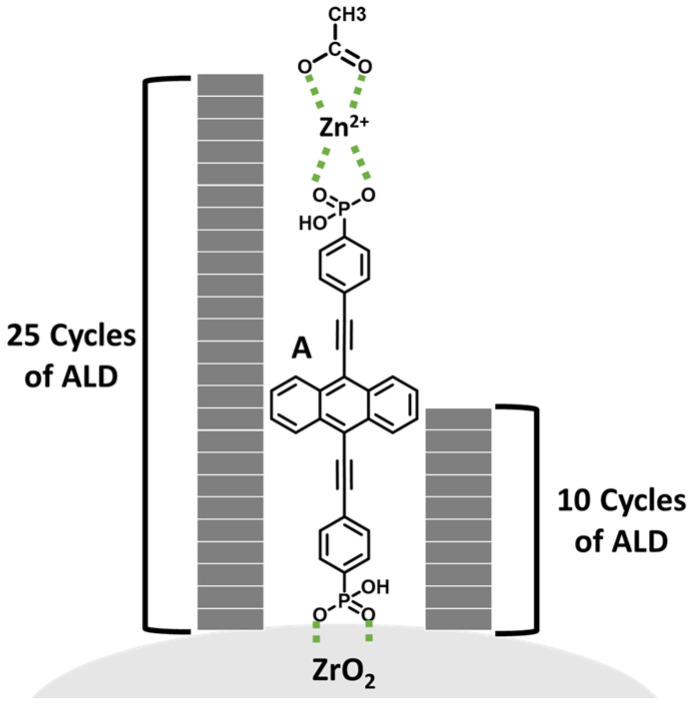
Depiction of ZrO_2_-**A** interface with the approximate thickness of 10 and 25 cycles of ALD (i.e., TMA/H_2_O) assuming complete Al_2_O_3_ layer growth at a rate of 1.1 Å per cycle.

Here we describe our effort using metal oxide overlayers generated via atomic layer deposition (ALD) to influence intermolecular interactions on a metal oxide surface. ALD typically involves alternating cycles of a transition metal precursor and oxidant for the self-limiting generation of uniform metal oxide layers [21]. Previously, it has been shown that low-temperature ALD is sufficiently gentle for the growth of metal oxide layers on molecule functionalized metal oxide surfaces [22,23]. This results in increased stability [24], improved catalytic activity [25], inhibited lateral hole hopping [26], and enhanced solar cell performance [22,23]. Here we use ALD to deposit aluminum oxide (Al_2_O_3_) on a ZrO_2_ surface functionalized with phosphonated 9,10-bis(phenylethynyl)anthracene (**A** in Figure 1) to investigate the impact of Al_2_O_3_ deposition on the photophysical properties and intermolecular interactions at a dye–metal oxide interface.

## 2. Results and Discussion

### 2.1. Surface Loading

#### 2.1.1. **A** on ZrO_2_

The dye–metal oxide system of interest for this study is ((anthracene-9,10-diylbis(ethyne-2,1-diyl))bis(4,1-phenylene))bis(phosphonic acid) (**A**) bound to a mesoporous ZrO_2_ surface (ZrO_2_-**A**) as depicted in Figure 1. **A** was selected as the chromophore because it was previously prepared in our lab for use in an upconversion solar cell [3]. Additionally, derivatives of the 9,10-bis(phenylethynyl)anthracene chromophores are known to undergo a variety of intermolecular processes including TTA-UC [3], singlet fission [4,27], and excimer formation [2,27]. ZrO_2_ was selected as the metal oxide substrate, as opposed to the more common TiO_2_ because its relatively high conduction band allows the investigation of the excited state dynamics in the absence of competitive electron injection into the substrate [28,29]. The preparation of mesoporous ZrO_2_ films is described in Section 3.2.

Dye loading was achieved by soaking the ZrO_2_ films in DMSO solutions of **A** at 90 °C. The surface coverage (Γ) with respect to loading time and concentration was monitored using UV-Vis spectroscopy and the results are shown in Figure S1. Γ was calculated using Equation (1) [30],

Γ = (*A*(λ_abs_)/ε(λ))/1000 (1)

where *A* and ε are the absorbance and extinction coefficient (7084 cm^2^ mol^−1^, Figure S2) for **A** at 475 nm, respectively. From fitting the concentration dependent isotherms [31], a maximum surface coverage (Γ_max_) of 240 nmol cm^−2^ was obtained for soaking in 250 µM **A**. For subsequent studies, the 100%, 50%, and 10% **A**-loaded films were obtained by soaking ZrO_2_ in a 250 µM DMSO solution of **A** for 5 min, 30 min, and 24 h, respectively, at 90 °C. 

#### 2.1.2. Zn(OAc)_2_ Treatment

**Figure 2:**
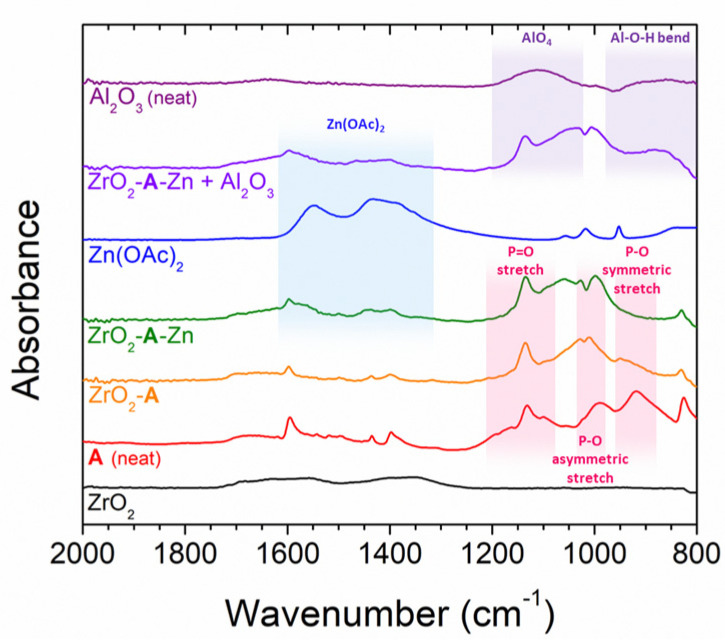
ATR-IR spectra of ZrO_2_, ZrO_2_-**A**, ZrO_2_-**A**-Zn, and ZrO_2_-**A**-Zn after 10 cycles of ALD (ZrO_2_-**A**-Zn + Al_2_O_3_) as well as neat powders of Al_2_O_3_, **A**, and Zn(OAc)_2_.

Trimethyl aluminum (TMA) from ALD treatments is known to react with hydroxyl groups of both the metal oxide surface and the phosphonate groups of surface bound molecules [32,33]. To hinder any possible reaction between TMA and **A**, we “capped” **A** using a Zn(OAc)_2_ treatment. That is, ZrO_2_-**A** slides were soaked in a 0.5 µM solution of Zn(OAc)_2_ in methanol for 2 h and the coordination was monitored using ATR-IR (Figure 2).

The ATR-IR spectrum of ZrO_2_-**A** has spectral contributions from both ZrO_2_ and **A**. The decrease in amplitude of the 1180 cm^−1^ and 920 cm^−1^ peaks of neat **A** is attributed to one of the two geometrically opposed phosphonate groups binding to the ZrO_2_ surface [34,35]. Following treatment of ZrO_2_-**A** with Zn(OAc)_2_ there is a shift of the 1010 cm^−1^ peak to 1000 cm^−1^ and a further decrease in the 920 cm^−1^ peak of **A**, as well as new contribution of acetate features at 1550 cm^−1^ and 1430 cm^−1^. These observations are consistent with the coordination of zinc acetate to the non-surface bound PO_3_H_2_ group of **A** (i.e., ZrO_2_-**A**-Zn) [34]. It is important to note that previous work has shown that zinc coordination has minimal impact on the photophysical properties of ZrO_2_-**A** [3].

#### 2.1.3. Al_2_O_3_ Overlayers

Al_2_O_3_ overlayers were deposited on ZrO_2_-**A**-Zn via ALD using alternating cycles of TMA and water (see Section 3.3 for details). Following ALD treatment of ZrO_2_-**A**-Zn the ATR-IR spectrum of the film (ZrO_2_-**A**-Zn + ALD in Figure 2) exhibits an additional AlO_4_ stretching feature at 880 cm^−1^ and Al-O-H bending feature at 1115 cm^−1^ that are consistent with the formation of Al_2_O_3_ [36]. The amplitude of the Al_2_O_3_ related IR features increase with increase with increasing cycles of ALD (Figure S4). Similarly, as measured by X-ray fluorescence (XRF), the amount of Al relative to Zr and Zn increases with increasing cycles of ALD (Figure S3, Table S1). Ten and twenty-five cycles of ALD were somewhat arbitrarily chosen for subsequent studies as to partially and completely surround **A**-Zn in Al_2_O_3_ as depicted in Figure 1. The height of the Al_2_O_3_ layers, relative to the molecule, is estimated from the well-established growth rate of 1.0–1.3 Å per cycle of TMA/H_2_O at 100 °C [37,38,39,40,41]. However, these deposition rates are for planar surfaces not functionalized with a molecule. However, the actual deposition rate and Al_2_O_3_ height in the ZrO_2_-**A**-Zn films is not known. Nevertheless, it can be concluded from ATR-IR and X-ray fluorescence (XRF) measurements that (1) Al_2_O_3_ is being formed and (2) the amount increases with the number of ALD cycles (Figures S3 and S4, Table S1). With that said, the above thickness estimate is not unreasonable, as related work with 80 cycles of TMA/H_2_O on dye-functionalized mesoporous metal oxide used TEM shows the expected overlayer thickness [42] and uniform distribution of Al_2_O_3_ in the mesoporous films [43].

### 2.2. Photophysical Properties of *ZrO_2_-**A***

Prior to investigating the impact of ALD on film photophysics, we measured the surface loading dependence on the absorption and emission properties of ZrO_2_-**A** in acetonitrile. ZrO_2_-**A** films with 100%, 50%, and 10% loading of **A** were compared with the properties of **A** in DMSO (i.e., sol’n).

**Figure 3:**
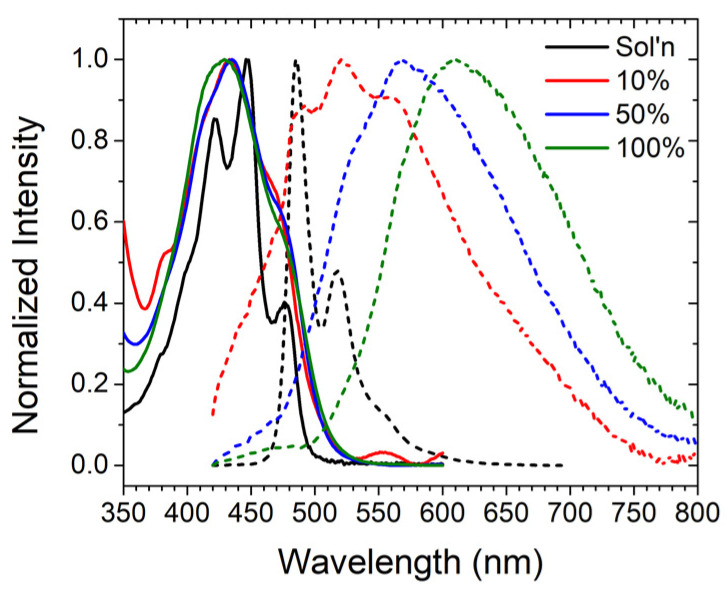
Absorbance (solid line) and emission (dotted line) spectra of **A** in DMSO and **A** on ZrO_2_ at 10%, 50%, and 100% surface loading. (λ_ex_ = 395 nm).

In contrast to the structured absorption observed for **A** in solution, there is notable peak broadening when bound to ZrO_2_. This behavior is common at dye–metal oxide interfaces and is typically attributed to inhomogeneous broadening due to variations in the local environment on the surface [44]. It is notable that there is minimal change in the energy of absorption and the spectra were similar regardless of surface loading (Figure 3).

Emission from **A** in solution is also highly structured with a maximum at 485 nm that is consistent with emission from the singlet excited state of 9,10-bis(phenylethynyl)anthracene [45]. For the 10%-loaded ZrO_2_-**A** sample, the 485 nm emission peak and vibrational structure are observed but are accompanied by a broad lower energy emission band from 500–800 nm. This new emission band becomes increasingly prominent for the 50%- and 100%-loaded films, with the latter being almost exclusively lower-energy emissions, with a peak at 610 nm. This feature has been observed previously for other solid-state bis(phenylethynyl)anthracene samples, and is attributed to excimer emission [27,46]. Interestingly, one would expect a homogeneously distributed 10%-loaded ZrO_2_-**A** film to exhibit only single molecule emission (i.e., with the molecules spaced out) but the presence of both monomer and excimer emission suggests a surface composed of both isolated **A** molecules and regions of at least two directly adjacent **A** molecules even with the most dilute sample measured here (i.e., 10% ZrO_2_-**A**).

The emission decay kinetics were monitored using time-correlated single photon counting, and the results are shown in Figure S6. Emission kinetics from **A** in DMSO were fit with a single exponential decay equation giving a lifetime of 2.6 ns at all wavelengths.

Likely due to inhomogeneities of the films as well as the multicomponent nature of the samples, decay kinetics from ZrO_2_-**A** could not be fit with a single exponential function. Similarly attempts at global analysis (i.e., singular value decomposition) failed to converge or provided physically unrealistic results. Consequently, the decays were fit with a biexponential function with the amplitude (*A*_x_) and lifetimes (τ_x_) of each component as well as the weighted average lifetime (τ_w_) summarized in Table 1. The 10%-loaded ZrO_2_-**A** film exhibits lifetimes of 3.7 ns at 500 nm and 10.4 ns at 600 nm, which are dominated by monomer and excimer emission, respectively. At increased surface loading, the lifetime increases, likely due to the increased contribution from the slower, low energy peak. These results are consistent with previous surface loading concentration dependent excimer formation in pyrenes [47,48,49,50].

**Figure 4:**
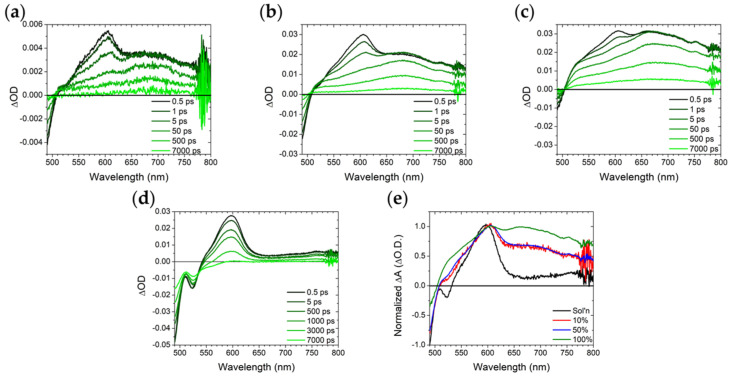
Transient absorption spectra from 0.5 to 7000 ps (black to light green) for ZrO_2_-**A** in MeCN with (**a**) 10%, (**b**) 50%, and (**c**) 100% loading as well as (**d**) **A** in DMSO; (**e**) 0.5 ps time slices for all samples normalized to the peak at 610 nm. (λ_ex_ = 475 nm).

The sub 7 ns excited-state dynamics for the samples were measured using transient absorption spectroscopy and the results are shown in Figure 4 with fitting parameters summarized in Table S2. For **A** in solution (Figure 4d), there is a ground state bleach below 540 nm and an excited state absorption (ESA) centered at 610 nm which has previously been assigned to the singlet excited state of 9,10-bis(phenylethynyl)anthracene [2,3]. The lifetime of these features (1.8 ns) is in reasonable agreement with the emission decay kinetics for the same sample (2.6 ns) further supporting the assignment.

When bound to the surface, all ZrO_2_-**A** exhibit the same ~610 nm ESA but also a new, broad ESA centered at ~670 nm (Figure 4a–c) that decays on comparable time scales as the excimer emission described above (5–10 ns). Myong et al. observed similar transient absorption features for a silyl-derivatized 9,10-bis(phenylethynyl)anthracene on an Al_2_O_3_ surface and attributed it to excimer formation [2]. For all three ZrO_2_-**A** samples there is a rapid decrease in the 610 nm monomer feature in <50 ps and a plateau followed by slow decay of >7 ns for the 670 nm excimer peak. The amplitude of the excimer peak, relative to monomer (Figure 4e), is highest on the 100%-loaded ZrO_2_-**A** samples, further highlighting the surface loading dependence on excimer formation. 

### 2.3. *ZrO_2_-**A**-Zn* with Al_2_O_3_

With the ZrO_2_-**A** photophysics established, we sought to understand the impact of Al_2_O_3_ overlayers on 50%- and 100%-loaded ZrO_2_-**A**-Zn samples. The samples were treated with 10 and 25 ALD cycles of TMA/H_2_O as described in Section 3.3. Interestingly, for the 100%-loaded ZrO_2_-**A** samples, the ALD treatment had minimal impact on the steady-state and time-resolved properties of the films (Figures S8–S11, Tables S3 and S4). Presumably either the high loading of **A** hinders the deposition of Al_2_O_3_, or deposition occurs but there are high-density regions of **A** that are largely uninfluenced by Al_2_O_3_ elsewhere. Given that the ATR-IR data support the formation of Al_2_O_3_, even on the 100%-loaded film, we are inclined to believe the latter. Regardless of the cause, we will focus the discussion below on the impact of ALD overlayers on the properties of the 50%-loaded ZrO_2_-**A**-Zn films.

**Figure 5:**
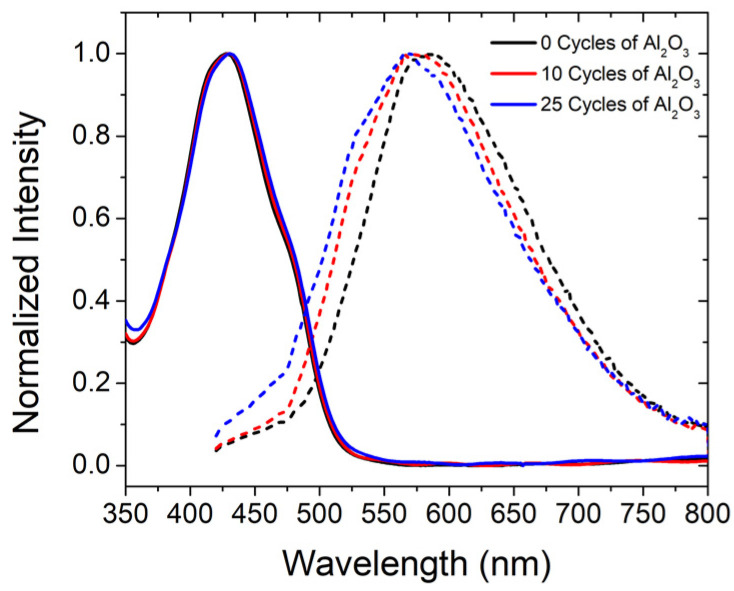
Absorbance (solid lines) and emission (dotted lines) of 50%-loaded ZrO_2_-**A**-Zn with 0 (black), 10 (red), and 25 (blue) cycles of TMA/H_2_O. (λ_ex_ = 395 nm).

The absorption and emission spectra of 50%-loaded ZrO_2_-**A**-Zn films with 0, 10, and 25 cycles of TMA/H_2_O are shown in Figure 5. ALD treatment had minimal impact on the amplitude or energy of the films’ absorption spectra. In contrast, with increasing ALD cycles, we see a larger contribution from the high energy side of the spectrum. The larger, high-energy contribution is accompanied by subtle vibrational features that are consistent with an increase in monomer emission at ~500 nm like that observed in for **A** in solution. Time resolved emission decays and fitting parameters are provided in Figure S12 and Table S5. Presumably due to the continued dominance of the excimer emission, ALD had minimal impact on the excited state decay kinetics.

**Figure 6:**
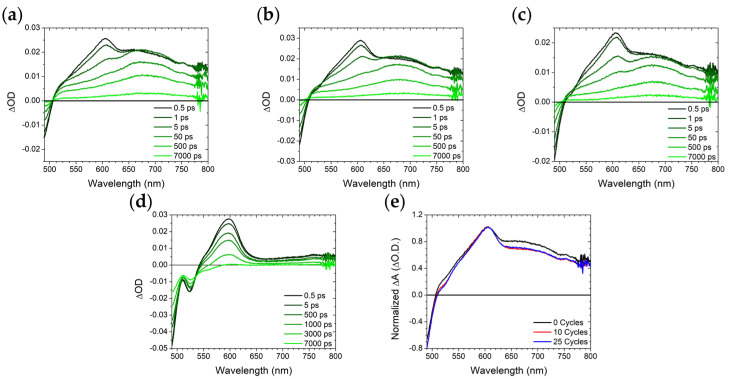
Transient absorption spectra from 0.5–7000 ps (black to light green) for 50%-loaded ZrO_2_-**A**-Zn in MeCN with (**a**) 0, (**b**) 10, and (**c**) 25 cycles TMA/H_2_O loading as well as (**d**) **A** in DMSO. (λ_ex_ = 475 nm); (**e**) the 0.5 ps time slices for all samples normalized to the peak at 610 nm.

Transient absorption measurements were performed on 50%-loaded ZrO_2_-**A**-Zn samples with 0, 10, and 25 cycles of TMA/H_2_O, and the results are shown in Figure 6 with fitting parameters summarized in Table S6. Similar to the untreated film (Figure 6a), the 10 and 25 cycle samples (Figure 6b,c) exhibit monomer and excimer ESA peaks at 610 and 670 nm, respectively. However, the relative amplitude of the excimer peak (Figure 6e) decreases for the ALD-treated films relative to the untreated film. The decrease in excimer ESA intensity combined with increased presence of monomer emission suggests that following ALD treatment, the deposition of Al_2_O_3_ plays at least some role in hindering excited state intermolecular interactions between surface bound **A** molecules. However, because it has minimal impact on the 100%-loaded samples and the 50%-loaded samples are still dominated by excimeric excited states, only a small fraction of the intermolecular interactions are impacted.

### 2.4. Photostability

**Figure 7:**
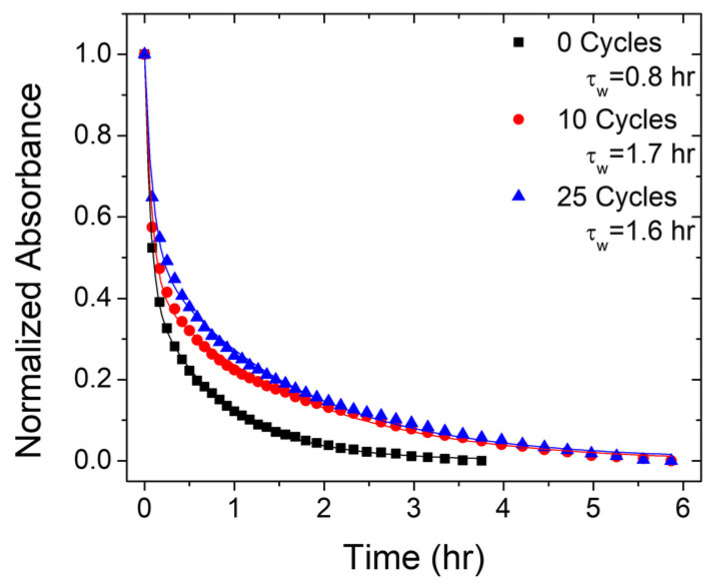
Absorption spectra changes for 50%-loaded ZrO_2_-**A**-Zn with 0 (black squares), 10 (red circles), and 25 (blue triangles) cycles of TMA/H_2_O monitored at 400 nm. Solid lines are the biexponential fits, and τ_w_ is the weighted average of those fits. (λ_ex_ = 455 nm).

Deposition of metal oxide overlayers has also been known to impact stability of surface-bound molecules [24]. To probe the photostability of the films studied here we irradiated films of 50%- and 100%-loaded ZrO_2_-**A**-Zn with 0, 10, and 25 cycles of TMA/H_2_O under 455 nm light and monitored the absorption change over a 6 h period (Figure S14). The decrease in absorption was similar at all wavelengths (i.e., there were no spectral shifts) and the intensity decrease at 400 nm can be seen in Figure 7.

Without ALD layers, the 100% films had a slower decrease in absorbance (τ_w_ = 1.0 h) than that of the 50%-loaded films (τ_w_ = 0.8 h). This observation is intriguing, as the 100% film absorbs more photons and might be expected to generate more heat via non-radiative pathways which could contribute to decomposition/desorption. Likewise, any bimolecular decay pathway would expect to be quicker on the more concentrated films. Presumably the intermolecular interactions of the 100% film help inhibit desorption or decomposition. For both the 50%-loaded and 100%-loaded samples, there is a general increase in stability of the films upon the addition of ALD overlayers. The stability enhancement is more pronounced for the 50%-loaded film, presumably due to either a less stable starting point or more uniform distribution of Al_2_O_3_ due to more surface availability. Regardless of the cause, the increased stability with ALD overlayers is in good agreement with prior results [24].

The decrease in absorbance is typically attributed to either decomposition or desorption of the molecules [51]. Anthracene derivatives are known to undergo photochemical reactions via dimerization at the 9,10 positions which is thermally reversible [52,53]. To probe possible dimerization, after 6 h of irradiation with 455 nm light, the ZrO_2_-**A**-Zn samples were placed in an oven at 90 °C overnight. No return of the anthracene absorption features was observed, suggesting that either dimerization is not the decomposition mechanism or the reaction is not thermally reversible on the surface. To investigate a possible desorption pathway, we measured the absorbance spectra of the solution after photolysis and the results are shown in Figure S15. The lack of 9,10-bis(phenylethynyl)anthracene absorption features rules out simple desorption of **A** from the surface. Instead, the new absorption peaks at 275 and 330 nm suggest a new byproduct with less conjugated core is being formed. One could envision either the photoreaction leading to detachment from the surface or the reaction generating a product that is less stable on the surface.

## 3. Materials and Methods

### 3.1. Chemicals and Materials

Molecule **A** was synthesized according to a previously published procedure [3]. Zinc acetate dihydrate (Alfa Aesar, Tewksbury, MA, USA), trimethyl aluminum (Strem Chemicals, Newburyport, MA, USA; prepackaged in a 50 mL Swagelok cylinder), ultrahigh purity water (Strem Chemicals, Newburyport, MA, USA; prepackaged in a 50 mL Swagelok cylinder), acetonitrile (Sigma-Aldrich, Milwaukee, WI, USA), methanol (Sigma-Aldrich, Milwaukee, WI, USA), dimethyl sulfoxide (DMSO, Alfa Aesar), ethanol (Koptec), hydrochloric acid (Sigma-Aldrich, Milwaukee, WI, USA), zirconium (IV) propoxide solution (Sigma-Aldrich, Milwaukee, WI, USA), nitric acid (Avantar, Radnor, PA, USA), polyethylene glycol bisphenol A epichlorohydrin copolymer (Sigma-Aldrich, Milwaukee, WI, USA) were purchased from their respective suppliers, in parentheses, and used as received. Non-conductive glass was purchased from Hartford Glass Co. (Hartford, CT, USA). Vac’n Fill Syringe (65209) and Melatonix films (1170-25) were purchased from Solaronix (Aubonne, Switzerland). Micro glass cover slides (18 × 18 mm) were purchased from VWR (Radnor, PA, USA).

### 3.2. Thin Film Sample Preparation

ZrO_2_ sol–gels were synthesized according to previous procedures [54]. Non-conductive glass was sonicated in HCl/EtOH (15:85; *v*/*v*) for 20 min and then in ethanol for 20 min and then dried. Nanoparticle ZrO_2_ films were prepared by doctorblading (1 layer 3M Scotch™ tape, Saint Paul, MN, USA)with ZrO_2_ sol–gel. The films were sintered at 430 °C for 15 min. The thin films were loaded as described in Section 2.1 and sealed with a piece of non-conductive glass using Melatonix thermoplastic heated to 150 °C with a home-built apparatus [55]. The sandwiched cells were then injected with acetonitrile in air using a Vac’n Fill Syringe (Solaronix, Aubonne, Switzerland). The hole was sealed using the same thermoplastic above and a glass coverslip heated with a soldering iron. The sandwiched cells were used for subsequent steady state emission, time resolved emission, and femtosecond transient absorption measurements.

### 3.3. Atomic Layer Deposition

Atomic layer deposition was performed with a Veeco Fiji G2 (Plainview, NY, USA) at 100 °C. The deposition recipe is as follows: trimethyl aluminum precursor spray 0.06 s, wait 15 s, H_2_O spray 0.06 s, wait 15 s. Samples underwent 10 or 10 + 15 (25 total) cycles of ALD.

### 3.4. Attenuated Total Reflectance–Fourier-Transform Infrared Spectroscopy (ATR-IR)

Attenuated total reflectance infrared spectra were collected with a Bruker Alpha FTIR spectrometer (SiC Glowbar source, DTGS detector) with a Platinum ATR QuickSnap sampling module single reflection diamond crystal (Bruker Alpha, Billerica, MA, USA). Spectra were acquired from 2000 cm^−1^ to 600 cm^−1^ at a resolution of 4 cm^−1^. All ATR-IR spectra are reported as absorbance with a blank versus atmosphere.

### 3.5. X-ray Fluorescence

Elemental composition for all samples were confirmed using X-ray Fluorescence (XRF) on a Panalytical Epsilon X-ray florescence analyzer (Malvern, UK; Cu Kα source).

### 3.6. Absorbance

Absorption spectra were collected with an Agilent 8453 UV-Visible photo diode array spectrophotometer (Agilent, Santa Clara, CA, USA). Solution samples were prepared by dissolving **A** in DMSO in a 1 × 1 cm quartz cuvette. Solid samples were prepared as described in Section 2.1. Before the sandwich cells were assembled, absorbance was measured by holding the thin film perpendicular to the beam path, with the film facing the source.

Loading isotherms were fit according to the Langmuir isotherm model in Equation (2) [31],

Γ = Γ_max_(*K_ad_* × [**A**])/(1 + *K_ad_* × [**A**]) (2)

where *K_ad_* is the adsorption equilibrium constant and Γ_max_ is the maximum surface coverage. The fitting parameters for **A** loaded at different concentrations and over time at 250 µM can be found in Figure S1.

### 3.7. Steady-State Emission

Steady state emission spectra were collected at room temperature on an Edinburgh FLS 980 fluorescence spectrometer (Livingston, UK). The samples were excited at 395 nm using a housed 450 W Xe lamp, passed through a single grating (1800 mm^−1^, 250 nm blaze) Czerny-Turner monochromator. Emissions from the sample were passed through a 420 nm long pass filter, then a single grating (1800 mm^−1^, 500 nm blaze) Czerny-Turner monochromator. Emission traces were detected by a Peltier-cooled Hamamatsu R928 photomultiplier tube (Bridgewater, NJ, USA). Spectra were processed with emission correction files on the Edinburgh software package (1.4.4, Build 2). 

Solution samples were prepared in deaerated DMSO in a 1 × 1 cm quartz cuvette. Solid samples were prepared as described in Section 2.1 and placed in an Edinburgh SM4 integrating sphere. The excitation slit was set to 20 nm and the emission slit was set to 3 nm, which remained unchanged for all samples. Emission was monitored from 420–800 nm.

### 3.8. Time Resolved Emission

Time resolved emission measurements were recorded at room temperature on an Edinburgh FLS980 fluorescence spectrometer (Livingston, UK). The samples were excited by an Edinburgh EPL-405 nm picosecond pulsed diode laser (Livingston, UK; 405 ± 10 nm, pulse width 57.6 ps) operated at 100 Hz. Emission traces were passed through a 420 long-pass filter followed by a single grating (1800 mm^−1^, 500 nm blaze) Czerny-Turner monochromator and were detected by a Peltier-cooled Hamamatsu R928 photomultiplier tube (Bridgewater, NJ, USA). Emission decay traces were acquired using time-correlated single photon counting (TCSPC; 1024 channels; 200 ns window) with data collection for 10,000 counts. The decay traces were fit with either a single or biexponential reconvolution fit using the Edinburgh software package (1.4.4, Build 2).

### 3.9. Femtosecond Transient Absorption

Transient absorption was measured with an Ultrafast Systems HELIOS FIRE transient absorption spectrometer (Ultrfast Systems, Sarasota, FL, USA) coupled to a Vitara-S Coherent Ti:sapphire laser (Coherent, Santa Clara, CA, USA). The signal was amplified using a 1 kHz Coherent Revolution-50 pump laser (Coherent, Santa Clara, CA, USA). The resulting 5 mJ pulse (100 fs FWHM, centered at 800 nm) was split into a pump and probe beam. The pump was directed through a Coherent OPerA Solo optical parametric amplifier (Coherent, Santa Clara, CA, USA), passed through a chopper to minimize scattering from the laser line, and passed through the sample with a resulting intensity of 74.1 ± 1.4 μJ/cm^−2^ at 475 nm. The probe beam was sent through a delay stage. A sapphire crystal (420–780 nm) produced a white light continuum from the probe beam. The pump and probe beams were then overlapped on the sample. The signal was collected by a CMOS detector.

For solution measurements, samples were prepared using deaerated DMSO in a 2 mm quartz cuvette with constant stirring throughout the measurement. Difference spectra and single wavelength kinetics were collected by averaging 3 scans, holding for 2 s, with an exponential point acquisition beginning with 0.001 ps steps for a total of 200 points.

Thin film samples were prepared as described in Section 2.1 and Section 3.2 and were mounted in a clamp stage. Difference spectra and single wavelength kinetics were collected from single scans, holding for 2 s, with an exponential point acquisition beginning with 0.001 ps steps for a total of 200 points. This was done for 2 separate spots on each thin film sample and the kinetics were averaged together. Doing multiple scans on the same area resulted in bleaching of the samples.

Data was processed using the Surface Xplorer (4.3.0) software package from Ultrafast Systems. On each day, a background scan of DMSO for solution samples and ZrO_2_ with no loaded A were performed. These scans were subtracted from the sample scans. Chirp corrections were performed afterwards. For solutions, decay traces were fit with mono-exponential equation. Thin film kinetics were fit using triexponential decays. The weighted average lifetimes (τ_w_) were calculated from the relative amplitude (*A_x_*) of each lifetime component (τ_x_) according to Equations (3) and (4) below.

*Φ*_x_ = (*A_x_*τ_x_)/((*A_x_*τ_x_) + (*A_y_*τ_y_) + (*A_z_*τ_z_)) (3)

τ_w_ = ((*Φ*_x_τ_x_^2^) + (*Φ*_y_τ_y_^2^) + (*Φ*_z_τ_z_^2^))/((*Φ*_x_τ_x_) + (*Φ*_y_τ_y_) + (*Φ*_z_τ_z_)) (4)

### 3.10. Photostability

A blue (455 nm, FWHM 18 nm, 38.2 mW) mounted high-power LED (Thorlabs, Inc., Newton, NJ, USA; M455L4) was used to illuminate samples. It was powered by a T-Cube LED driver (Thorlabs, Inc., Newton, NJ, USA; LEDD1B) set to maximum power. The LED was placed perpendicular to the beam path of an Agilent 8543 UV-Visible photo diode array spectrometer (Santa Clara, CA, USA). Thin films of ZrO_2_-**A**-Zn with and without ALD treatment were held at a 45° angle in a 1 cm quartz cuvette with 2.5 mL MeCN. The samples were positioned at the intersection of the LED and spectrometer light paths. The absorption spectrum (350–600 nm) of the film was collected every 300 s for the first 3600 s and then with a 5% time increase in sampling time up to 21,600 s. The incident light power was measured by a thermopile detector (Newport Corp, Irvine, CA, USA; 1918-C meter, 818P-020-12 detector).

## 4. Conclusions

In this report, we investigated the impact of surface loading and ALD overlayers on the photophysical properties of a 9,10-bis(phenylethynyl)anthracene bound to a metal oxide surface. While surface loading density had no impact on the absorption spectra of the films, there was an increase in excimer features with surface loading as observed by both emission and transient absorption. The addition of ALD overlayers of Al_2_O_3_ resulted in a small decrease in excimer formation, but the emission and transient absorption spectra were still dominated by excimer features. Why the impact of ALD was so subtle is not obvious to us. One could envision the intermolecular interactions being so strong as to hinder the deposition of Al_2_O_3_ between molecular pairs. However, the minimal change in the absorption spectra across all samples suggests that the ground state interaction in not particularly strong. Instead, the electronic structure change in the excited state facilitates intermolecular interactions which are favorable at high surface loadings but slightly hindered after ALD treatment. Consequently, while we initially hoped that ALD would have a more pronounced impact on excimer formation (e.g., completely hinder it), these results do highlight the difficulty and subtlety in controlling these interactions on a surface.

## Figures and Tables

**Table 1:** Emission decay fitting parameters at 500 and 600 nm for **A** in DMSO (sol’n) and ZrO_2_-**A** at 10%, 50%, and 100% loading in acetonitrile. Error bars are the standard deviation of three independent measurements. (λ_ex_ = 405 nm).

Sample	500 nm					600 nm				
	*A*_1_	τ_1_ (ns)	*A*_2_	τ_2_ (ns)	τ_w_ (ns) ^a^	*A*_1_	τ_1_ (ns)	*A*_2_	τ_2_ (ns)	τ_w_ (ns) ^a^
Sol’n	-	-	-	-	2.6 ± 0.1 ^b^	-	-	-	-	2.7 ± 0.1 ^b^
10%	0.65	0.3	0.017	4.2	3.7 ± 0.8	0.30	0.7	0.023	10.9	10.4 ± 0.3
50%	0.94	0.2	0.008	5.8	5.2 ± 0.5	0.30	0.8	0.027	11.8	11.2 ± 0.5
100%	0.50	0.3	0.003	6.7	5.4 ± 0.5	0.32	0.6	0.029	9.2	8.8 ± 0.9

^a^ From the weighted average of the biexponential fit parameters. ^b^ from the single exponential fit.

## Data Availability

Data can be made available upon request.

## Supplementary Materials

The following supporting information can be downloaded at: https://www.mdpi.com/article/10.3390/molecules28124835/s1, Figure S1: Loading Isotherms of ZrO_2_-**A** in DMSO; Figure S2: Extinction Coefficient Calculation (λ = 475 nm); Table S1: Ratio of Elements in ZrO_2_-**A**-Zn + Al_2_O_3_ Samples; Figure S3: X-ray Fluorescence Spectra of ZrO_2_-**A**-Zn + Al_2_O_3_; Figure S4: ATR-IR of ZrO_2_-**A**-Zn + Al_2_O_3_; Figure S5: Raw Absorption Spectra at Different Surface Loading; Figure S6: Emission Decay Traces at Different Surface Loading; Figure S7: Transient Absorption Decay Traces at Different Surface Loading; Table S2: Transient Absorption Decay Fitting Parameters for ZrO_2_-**A** at Different Surface Loading; Figure S8: Absorbance and Emission of 100% ZrO_2_-**A**-Zn + Al_2_O_3_; Figure S9: Emission Decay Traces of 100% ZrO_2_-**A**-Zn + Al_2_O_3_; Table S3: Emission Decay Fitting Parameters for ZrO_2_-**A**-Zn + Al_2_O_3_; Figure S10: fsTA Spectra of 100% ZrO_2_-**A**-Zn + Al_2_O_3_; Figure S11: Transient Absorption Decay Traces of 100% ZrO_2_-**A**-Zn + Al_2_O_3_; Table S4: Transient Absorption Decay Fitting Parameters for 100% ZrO_2_-**A**-Zn + Al_2_O_3_; Figure S12: Emission Decay Traces of 50% ZrO_2_-**A**-Zn + Al_2_O_3_; Table S5: Emission Decay Fitting Parameters for 50% ZrO_2_-**A**-Zn + Al_2_O_3_; Figure S13: Transient Absorption Decay Traces of 50% ZrO_2_-**A**-Zn + Al_2_O_3_; Table S6: Transient Absorption Decay Fitting Parameters for 50% ZrO_2_-**A**-Zn + Al_2_O_3_; Figure S14: Absorbance Spectra Changes in ZrO_2_-**A**-Zn + Al_2_O_3_; Table S7: Absorption Decrease Fitting Parameters of ZrO_2_-**A**-Zn + Al_2_O_3_; Figure S15: Absorption Spectra Changes for 100%-Loaded ZrO_2_-**A**-Zn + Al_2_O_3_ at 400 nm; Figure S16: 50%-Loaded ZrO_2_-**A**-Zn + Al_2_O_3_ Before and After Irradiation.

## Sample Availability

Samples are available upon request.
