# Supplementary material for: Influence of Al2O3 Overlayers on Intermolecular Interactions between Metal Oxide Bound Molecules
Source: Molecules. 2023 Jun 17;28(12):4835. doi: 10.3390/molecules28124835 (PMC10301487; doi:10.3390/molecules28124835)
Supplement: Supplementary file 1 [file molecules-28-04835-s001.zip › molecules-2402380-supplementary.pdf]

Influence of Al_2O_3 Overlayers on Intermolecular Interactions between Metal Oxide Bound Molecules

–Supplemental Information–

Erica S. Knorr, Cody T. Basquill, Isabella A. Bertini, Ashley Arcidiacono, Drake Beery, Jonathan P. Wheeler, J. S. Raaj Vellore Winfred, Geoffrey F. Strouse and Kenneth Hanson

Figure S1. Loading Isotherms of ZrO_2-**A** in DMSO………………………………………………..……….. **1**

Figure S2. Extinction Coefficient Calculation (λ=475 nm)…………………………………….……………… **1**

Table S1. Ratio of Elements in ZrO_2-**A**-Zn + Al_2O_3 Samples……………………………………..……… **2**

Figure S3. X-Ray Fluorescence Spectra of ZrO_2-**A**-Zn + Al_2O_3……………………………...………..… **2**

Figure S4. ATR-IR of ZrO_2-**A**-Zn + Al_2O_3………………………………………….……………………… **3**

Figure S5. Raw Absorption Spectra at Different Surface Loading……………………...……………… **3**

Figure S6. Emission Decay Traces at Different Surface Loading……………………….………………… **4**

Figure S7. Transient Absorption Decay Traces at Different Surface Loading……………...…..……… **4**

Table S2. Transient Absorption Decay Fitting Parameters for ZrO_2-**A** at Different Surface Loading… **4**

Figure S8. Absorbance and Emission of 100% ZrO_2-**A**-Zn + Al_2O_3……………………….……….…… **5**

Figure S9. Emission Decay Traces of 100% ZrO_2-**A**-Zn + Al_2O_3…………………….……...……...… **5**

Table S3. Emission Decay Fitting Parameters for 100% ZrO_2-**A**-Zn + Al_2O_3……………….………… **6**

Figure S10. fsTA Spectra of 100% ZrO_2-**A**-Zn + Al_2O_3………………..………………………..………… **6**

Figure S11. Transient Absorption Decay Traces of 100% ZrO_2-**A**-Zn + Al_2O_3…………….…..……..… **7**

Table S4. Transient Absorption Decay Fitting Parameters for 100% ZrO_2-**A**-Zn + Al_2O_3………..…..… **7**

Figure S12. Emission Decay Traces of 50% ZrO_2-**A**-Zn + Al_2O_3……………..…………….……..…..…… **7**

Table S5. Emission Decay Fitting Parameters for 50% ZrO_2-**A**-Zn + Al_2O_3……………………….…... **8**

Figure S13. Transient Absorption Decay Traces of 50% ZrO_2-**A**-Zn + Al_2O_3………….…………..…… **8**

Table S6. Transient Absorption Decay Fitting Parameters for 50% ZrO_2-**A**-Zn + Al_2O_3……..……..… **8**

Figure S14. Absorbance Spectra Changes of ZrO_2-**A**-Zn + Al_2O_3…….…………………….………….. **9**

Table S7. Absorption Decrease Fitting Parameters of ZrO_2-**A**-Zn + Al_2O_3………………...…...……… **9**

Figure S15. Absorption Spectra Changes for 100% Loaded ZrO_2-**A**-Zn + Al_2O_3 at 400 nm……………... **10**

Figure S16. 50% Loaded ZrO_2-**A**-Zn + Al_2O_3 Before and After Irradiation……..……………………….. **10**

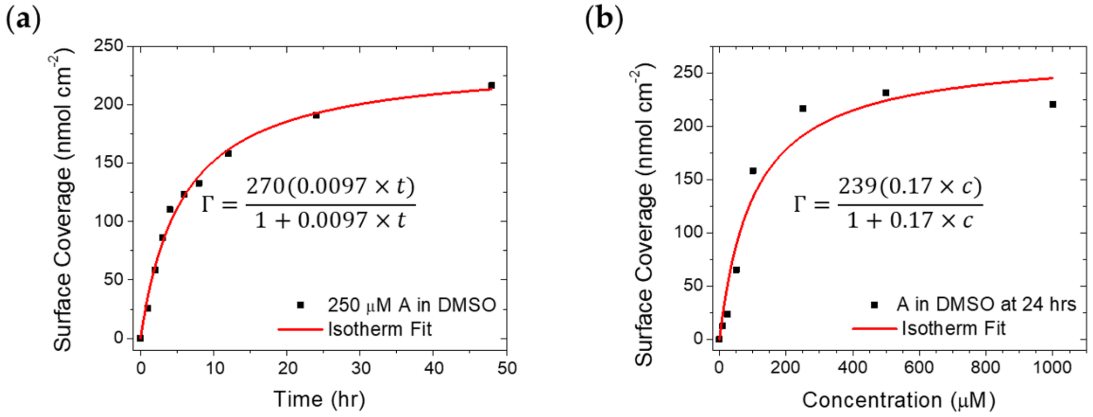

Figure S1. Isotherm loading for ZrO_2-**A** in DMSO (a) at 250 μM **A** in DMSO from 0-48 hours and (b) **A** in DMSO at concentrations of 0-1000 μM after 48 hours. Red lines are fits to the isotherm equation (Γ = surface coverage, c = concentration, and t = time).

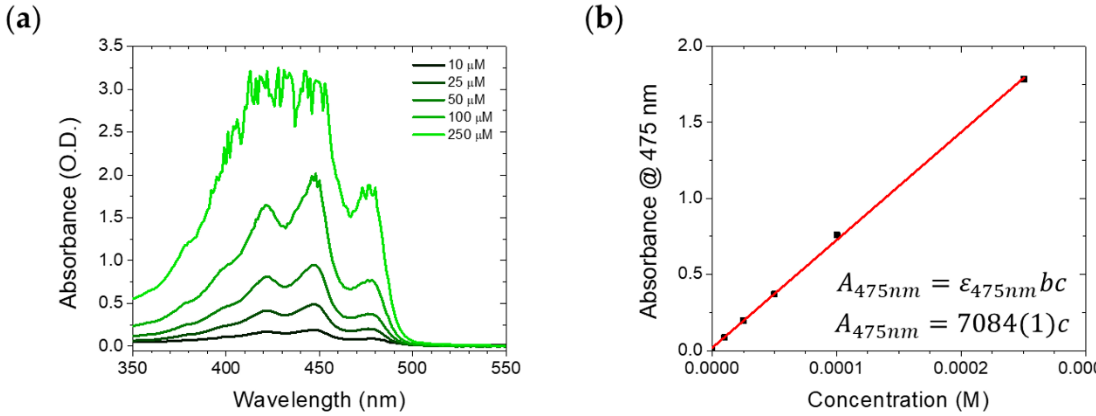

Figure S2. (a) Absorption spectra of **A** in DMSO at various concentrations; (b) Extinction coefficient Beer-Lambert plot at 475 nm for **A** in DMSO.

Table S1. Percent and normalized ratio (with Zr at 100) of Zr, Zn, and Al obtained via XRF of samples of 50% loaded ZrO_2-**A**-Zn with 0, 10, and 25 cycles of TMA/H_2O.

	**0 Cycles**		**10 Cycles**		**25 cycle**	
**Element**	**Amount (%)**	**Norm. Ratio**	**Amount (%)**	**Norm. Ratio**	**Amount (%)**	**Norm. Ratio**
Zr	91.9	100	83.9	100	80.3	100
Zn	1.98	2.15	1.8	2.15	1.7	2.12
Al	6.1	6.64	14.2	16.9	17.9	17.9

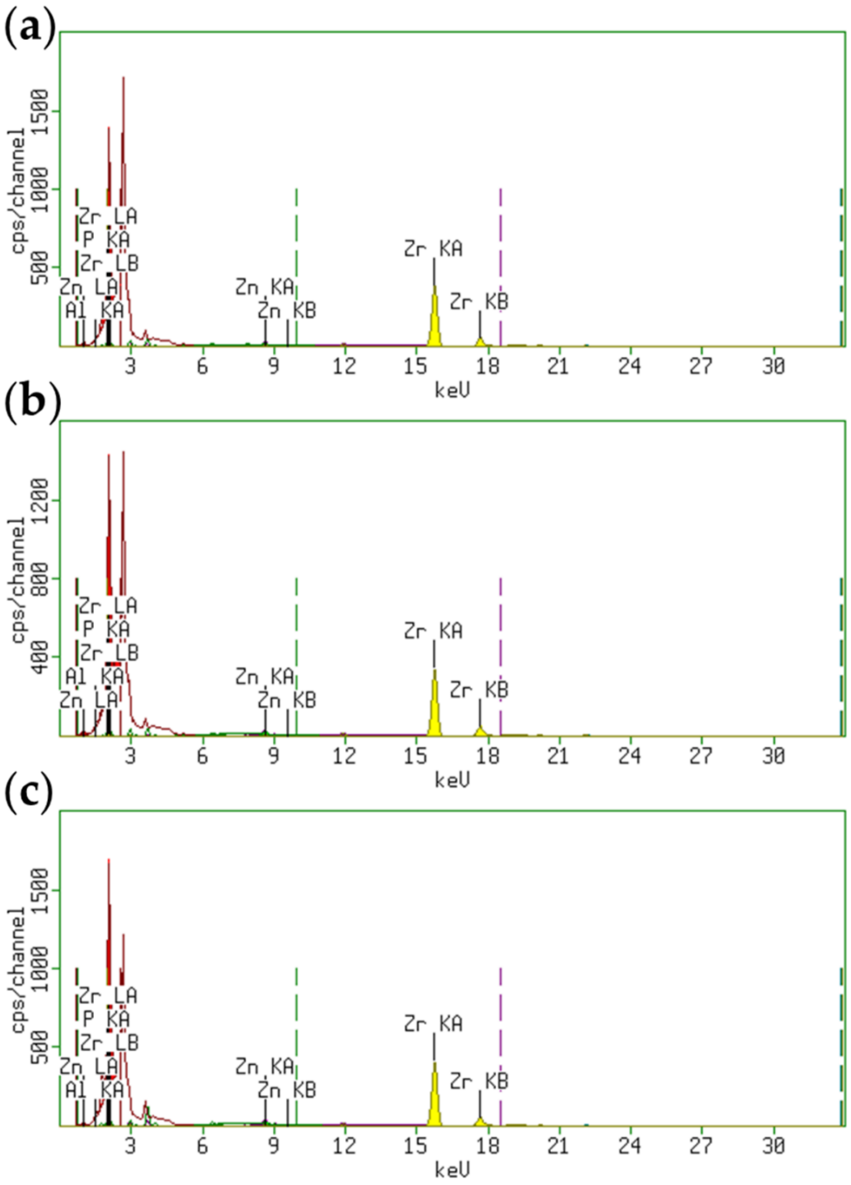

Figure S3. X-Ray Fluorescence spectra for ZrO_2-**A**-Zn after (**a**) 0, (**b**) 10, and (**c**) 25 cycles of TMA/H_2O.

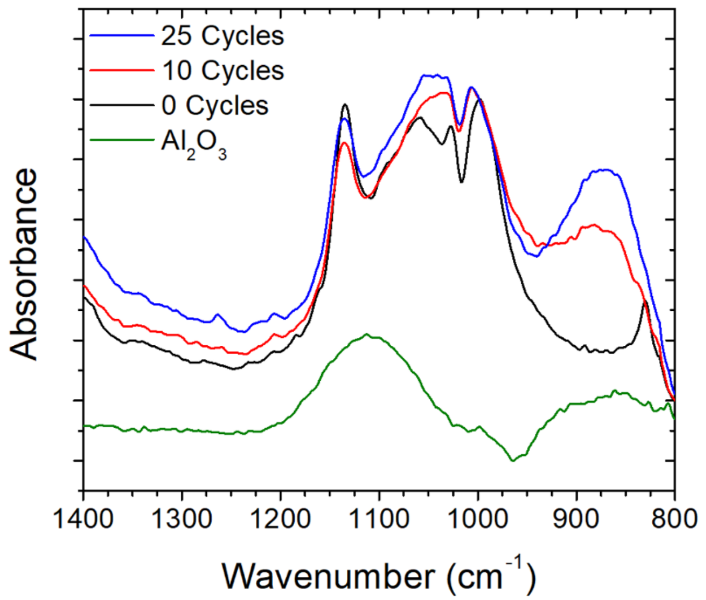

Figure S4. ATR-IR spectra of ZrO_2-**A**-Zn after 0 (black), 10 (red), and 25 (blue) cycles of TMA/H_2O as well as neat powder of Al_2O_3 (green). ZrO_2-**A**-Zn samples were normalized at 998 cm^{-1}.

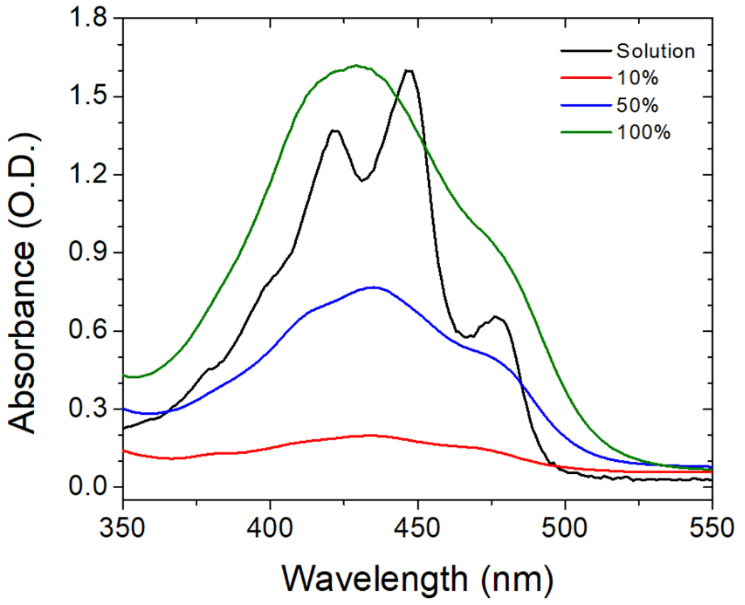

Figure S5. Absorption spectra of **A** in DMSO and ZrO_2-**A** at 10%, 50%, and 100% loading.

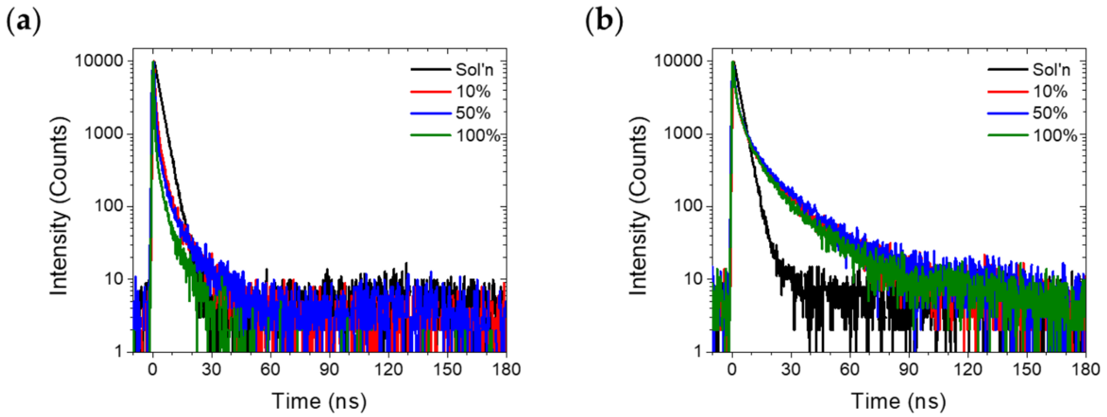

Figure S6. Emission decay traces at (a) 500 nm and (b) 600 nm for **A** in DMSO and ZrO_2-**A** at 10%, 50%, and 100% loading in acetonitrile.(λ_{ex}=405 nm)

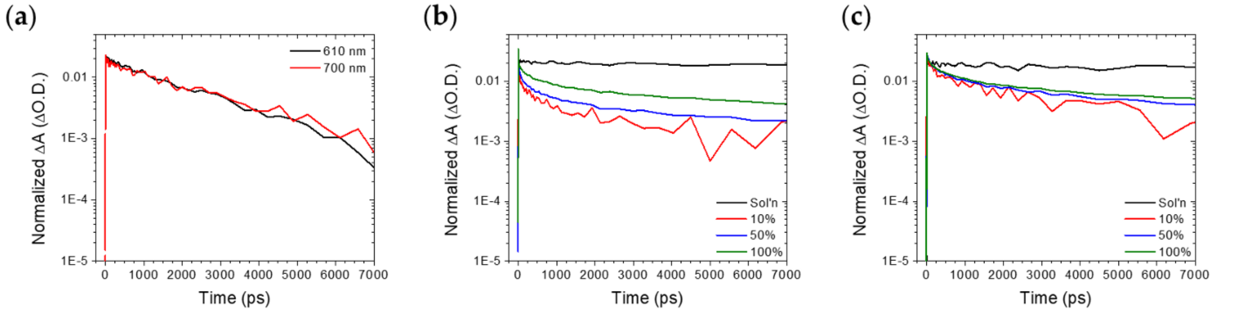

Figure S7. Transient absorption decay traces for (a) **A** in DMSO at 610 nm and 700 nm; Transient absorption decay traces at (b) 610 nm and (c) 700 nm for **A** in DMSO and ZrO_2-**A** at 10%, 50%, and 100% loading in acetonitrile. (λ_{ex}=475 nm)

Table S2. Transient absorption decay fitting parameters at 610 and 700 nm for **A** in DMSO (sol'n) and ZrO_2-**A** in acetonitrile at 10%, 50%, and 100% loading. Error bars are the standard deviation of three independent measurements. (λ_{ex}=475 nm)

Sample	610 nm							700 nm						
	A_1	τ_1 (ps)	A_2	τ_2 (ps)	A_3	τ_3 (ps)	τ_w (ps)[a]	A_1	τ_1 (ps)	A_2	τ_2 (ps)	A_3	τ_3 (ps)	τ_w (ps)[a]
Sol'n	-	-	-	-	-	-	1780±50[b]	-	-	-	-	-	-	-
10%	0.002	1.2	0.007	32	0.0012	2415	2400±700	0.0008	20	0.002	186	0.0012	4715	4700±400
50%	0.018	2.5	0.015	78	0.009	3199	3200±400	0.009	61	0.01	527	0.008	7489	7400±800
100%	0.01	2.6	0.010	144	0.010	6133	6130±190	0.008	50	0.011	541	0.010	9501	9470±170

[a] From the weighted average of the triexponential fit parameters. [b] from the single exponential fit.

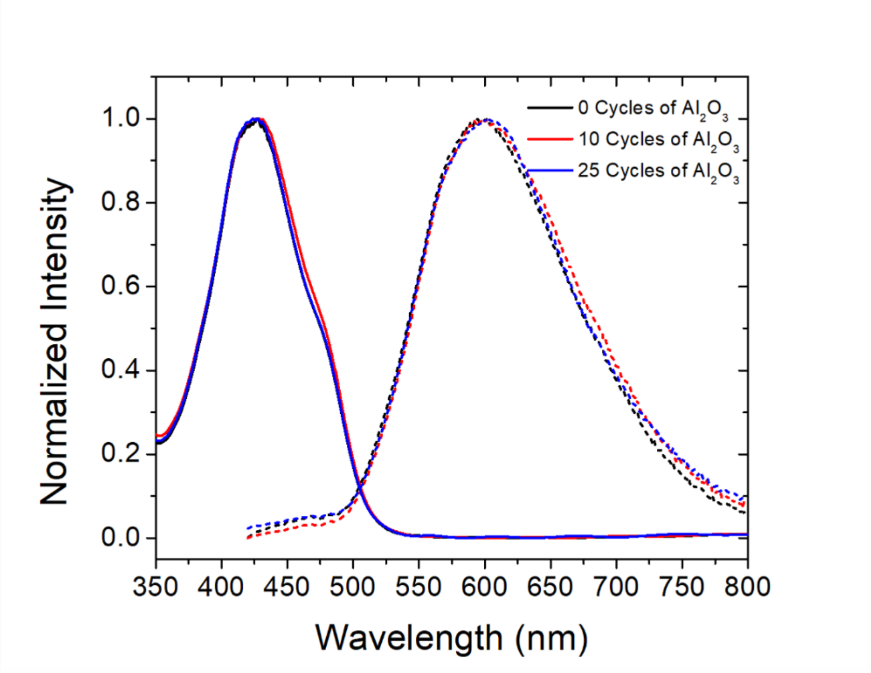

Figure S8. Absorbance (solid lines) and emission (dotted lines) of 100% loaded ZrO_2-**A**-Zn with 0 (black), 10 (red), and 25 (blue) cycles of TMA/H_2O (λ_{ex}=395 nm).

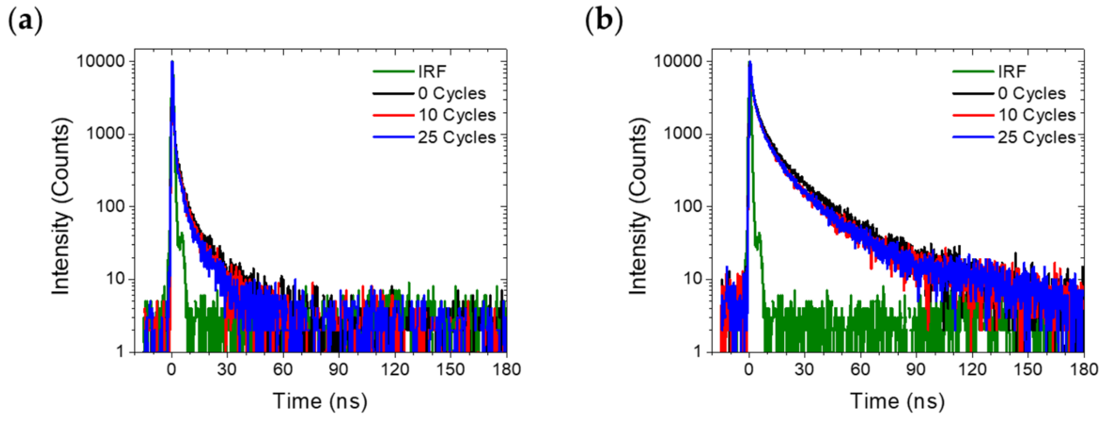

Figure S9. Emission decay traces at (a) 500 nm and (b) 600 nm for 100% loaded ZrO_2-**A**-Zn with 0 (black), 10 (red), and 25 (blue) cycles of TMA/H_2O in acetonitrile. (λ_{ex}=405 nm)

Table S3. Emission decay fitting parameters at 500 and 600 nm for **A** in DMSO (sol'n) and 100% loaded ZrO_2-**A**-Zn with 0, 10, and 25 cycles of TMA/H_2O in acetonitrile. Error bars are the standard deviation of three independent measurements. (λ_{ex}=405 nm)

Sample	500 nm					600 nm				
	A_1	τ_1 (ns)	A_2	τ_2 (ns)	τ_w (ns)[a]	A_1	τ_1 (ns)	A_2	τ_2 (ns)	τ_w (ns)[a]
Sol'n	-	-	-	-	2.6±0.1[b]	-	-	-	-	2.7±0.1[b]
0 cycles	0.91	0.1	0.005	8.1	7.8±0.2	0.30	0.7	0.030	10.9	10.5±1.7
10 cycles	0.75	0.2	0.004	7.0	6.4±0.7	0.29	0.7	0.027	11.3	10.8±0.6
25 cycles	0.99	0.1	0.005	7.1	6.7±0.4	0.24	0.99	0.026	11.9	11.24±0.98

[a] From the weighted average of the biexponential fit parameters. [b] from the single exponential fit.

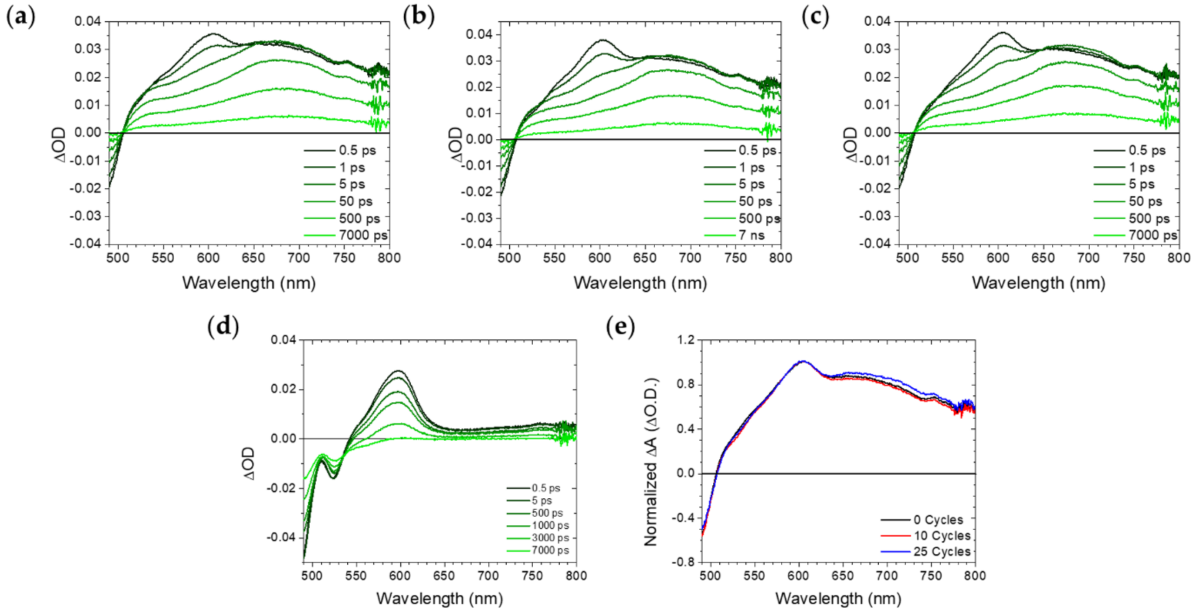

Figure S10. Transient absorption spectra from 0.5-7000 ps (black to light green) for 100% loaded ZrO_2-**A**-Zn in MeCN with (**a**) 0, (**b**) 10, and (**c**) 25 cycles TMA/H_2O loading as well as (**d**) A in DMSO (λ_{ex}=475 nm). (**e**) The 0.5 ps time slices for all samples normalized to the peak at 610 nm.

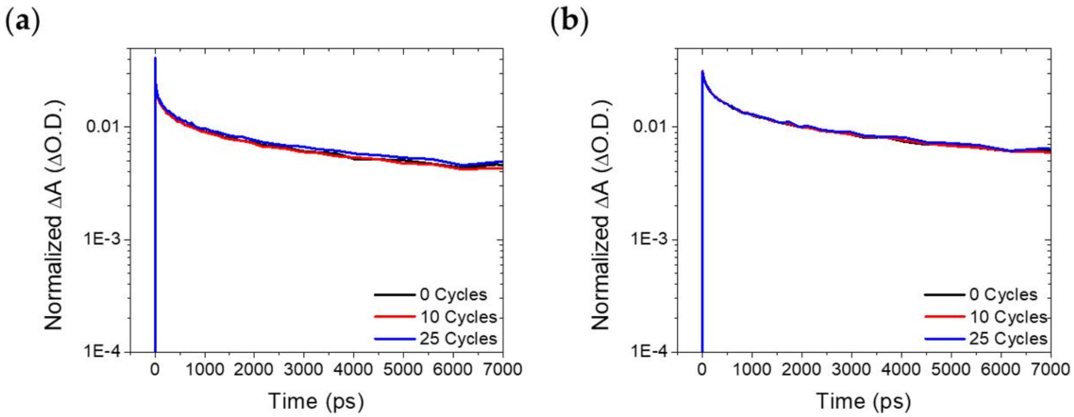

Figure S11. Transient absorption decay traces (λ_{ex}=475 nm) for 100% loaded ZrO_2-**A**-Zn with 0 (black), 10 (red), and 25 (blue) cycles of TMA/H_2O at (a) 610 nm and (b) 700 nm.

Table S4. Transient absorption decay fitting parameters at 610 and 700 nm for **A** in DMSO (sol'n) and 100% loaded ZrO_2-**A**-Zn with 0, 10, and 25 cycles of TMA/H_2O in acetonitrile. Error bars are the standard deviation of three independent measurements. (λ_{ex}=475 nm)

Sample	610 nm							700 nm							
	A_1	τ_1 (ps)	A_2	τ_2 (ps)	A_3	τ_3 (ps)	τ_w (ps)[a]	A_1	τ_1 (ps)	A_2	τ_2 (ps)	A_3	τ_3 (ps)	τ_w (ps)[a]
Sol'n	-	-	-	-	-	-	1780±50[b]	-	-	-	-	-	-	-
0 cycles	0.014	2.7	0.012	135	0.011	5521	5500±500	0.009	79	0.010	644	0.011	9951	9915±96
10 cycles	0.015	2.6	0.011	125	0.011	5324	5300±400	0.009	73	0.010	651	0.011	9882	9800±900
25 cycles	0.013	2.6	0.010	125	0.010	5648	5600±200	0.008	59	0.010	577	0.011	10087	10000 ±1600

[a] From the weighted average of the triexponential fit parameters. [b] from the single exponential fit.

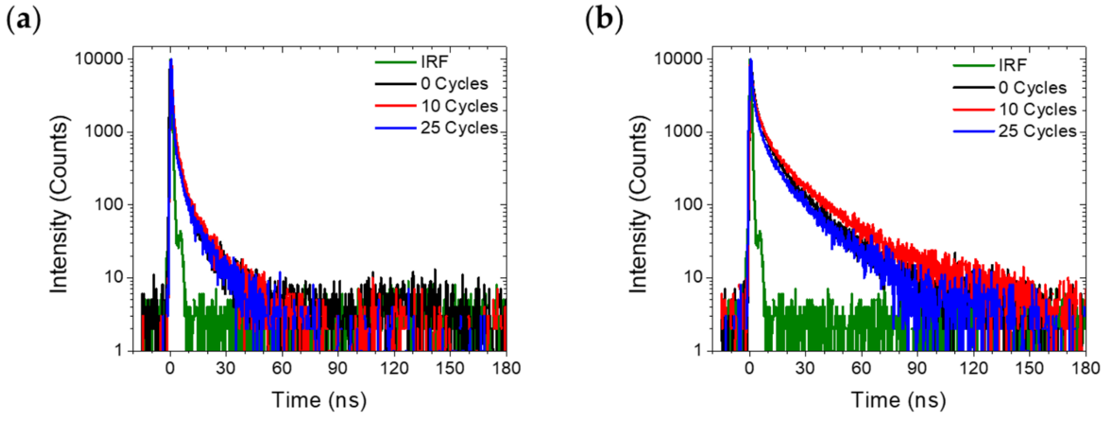

Figure S12. Emission decay traces at (a) 500 nm and (b) 600 nm for 50% loaded ZrO_2-**A**-Zn with 0 (black), 10 (red), and 25 (blue) cycles of TMA/H_2O in acetonitrile. (λ_{ex}=405 nm)

Table S5. Emission decay fitting parameters at 500 and 600 nm for **A** in DMSO (sol'n) and 50% loaded ZrO_2-**A**-Zn with 0, 10, and 25 cycles of TMA/H_2O in acetonitrile. Error bars are the standard deviation of three independent measurements. (λ_{ex}=405 nm)

Sample	500 nm					600 nm				
	A_1	τ_1 (ns)	A_2	τ_2 (ns)	τ_w (ns)[a]	A_1	τ_1 (ns)	A_2	τ_2 (ns)	τ_w (ns)[a]
Sol'n	-	-	-	-	2.6±0.1[b]	-	-	-	-	2.7±0.1[b]
0 cycles	0.59	0.3	0.017	52	4.7±1.4	0.33	0.7	0.027	10.6	10.1±0.4
10 cycles	0.48	0.4	0.008	7.4	6.5±0.6	0.25	0.9	0.025	12.9	12.4±1.6
25 cycles	0.58	0.4	0.020	5.6	5±2	0.25	1.0	0.023	12.2	11.4±0.3

[a] From the weighted average of the biexponential fit parameters. [b] from the single exponential fit.

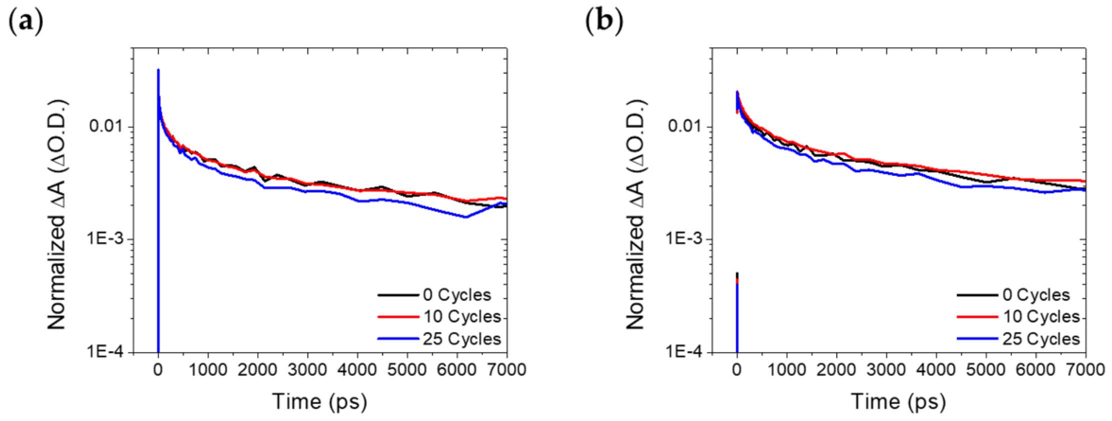

Figure S13. Transient absorption decay traces at (a) 610 nm and (b) 700 nm for 50% loaded ZrO_2-**A**-Zn with 0 (black), 10 (red), and 25 (blue) cycles of TMA/H_2O. (λ_{ex}=475 nm)

Table S6. Transient absorption decay fitting parameters at 610 and 700 nm for **A** in DMSO (sol'n) and 50% loaded ZrO_2-**A**-Zn with 0, 10, and 25 cycles of TMA/H_2O in acetonitrile. Error bars are the standard deviation of three independent measurements. (λ_{ex}=475 nm)

Sample	610 nm							700 nm						
	A_1	τ_1 (ps)	A_2	τ_2 (ps)	A_3	τ_3 (ps)	τ_w (ps)[a]	A_1	τ_1 (ps)	A_2	τ_2 (ps)	A_3	τ_3 (ps)	τ_w (ps)[a]
Sol'n	-	-	-	-	-	-	1780±50[b]	-	-	-	-	-	-	-
0 cycles	0.013	1.8	0.009	79	0.007	4022	4000±700	0.007	41	0.008	445	0.008	8056	8000±700
10 cycles	0.014	2.8	0.010	98	0.008	4291	4300±400	0.007	54	0.009	501	0.008	8689	8700±900
25 cycles	0.012	2.5	0.009	85	0.006	3800	3800±500	0.006	32	0.008	365	0.007	7192	7200±300

[a] From the weighted average of the triexponential fit parameters. [b] from the single exponential fit.

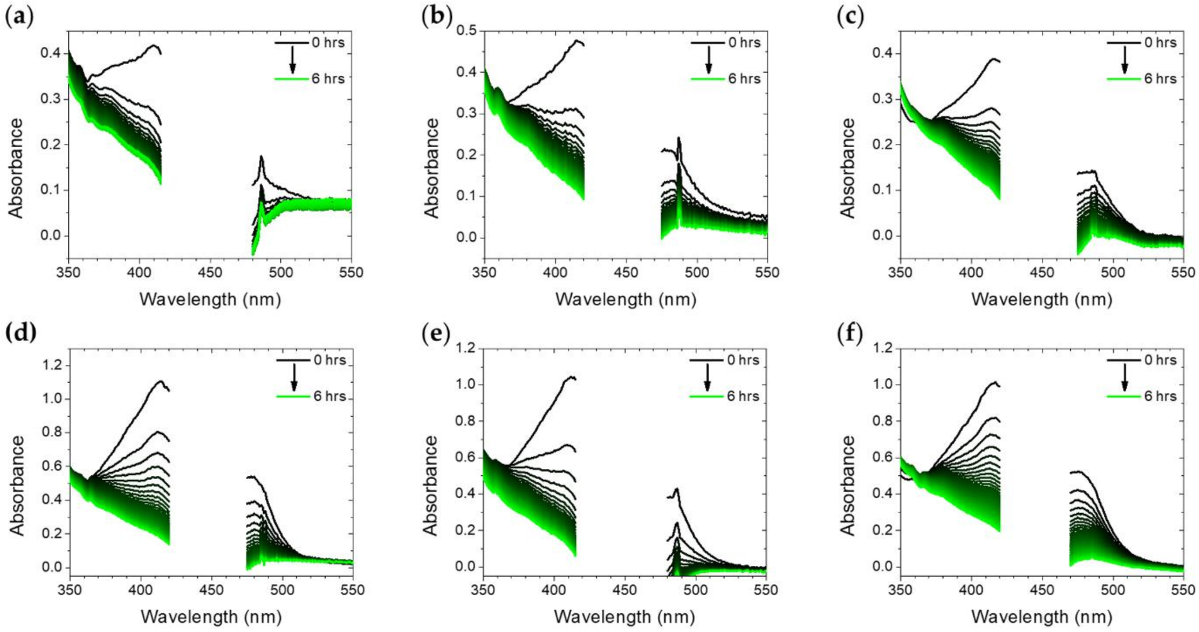

Figure S14. Absorption spectra changes of 50% (a-c) and 100% (d-f) loaded ZrO_2-**A**-Zn with 0 (a,d), 10 (b,e), and 25 (c,f) cycles of TMA/H_2O in MeCN under 455 nm irradiation.

Table S7. Biexponential fitting parameters for the absorption decrease at 400 nm for 50% loaded and 100% loaded ZrO_2-**A**-Zn with 0, 10, and 25 cycles of TMA/H_2O in acetonitrile under 455 nm irradiation.

Sample	50% Loaded					100% Loaded				
	A_1	τ_1 (hr)	A_2	τ_2 (hr)	τ_w (hr)[a]	A_1	τ_1 (hr)	A_2	τ_2 (hr)	τ_w (hr)[a]
0 cycles	0.58	0.06	0.42	0.81	0.8	0.53	0.14	0.45	1.01	1.0
10 cycles	0.57	0.08	0.43	1.69	1.7	0.65	0.13	0.33	1.28	1.3
25 cycles	0.091	0.09	0.096	1.58	1.6	0.46	0.18	0.085	1.66	1.6

[a] From the weighted average of the biexponential fit parameters. [b] from the single exponential fit.

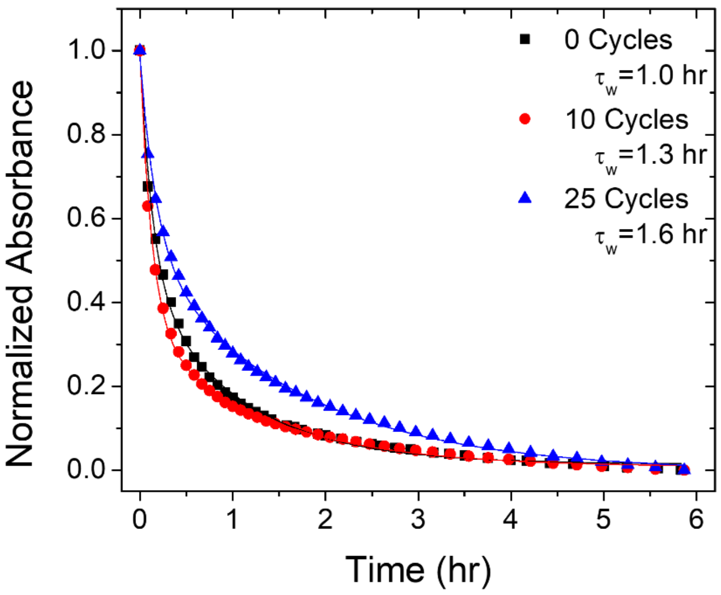

Figure S15. Absorption spectra changes for 50% loaded ZrO_2-A-Zn with 0 (black squares), 10 (red circles), and 25 (blue triangles) cycles of TMA/H_2O monitored at 400 nm. Solid lines are the biexponential fits and t_w is the weighted average of those fits. (λ_{ex}=455 nm)

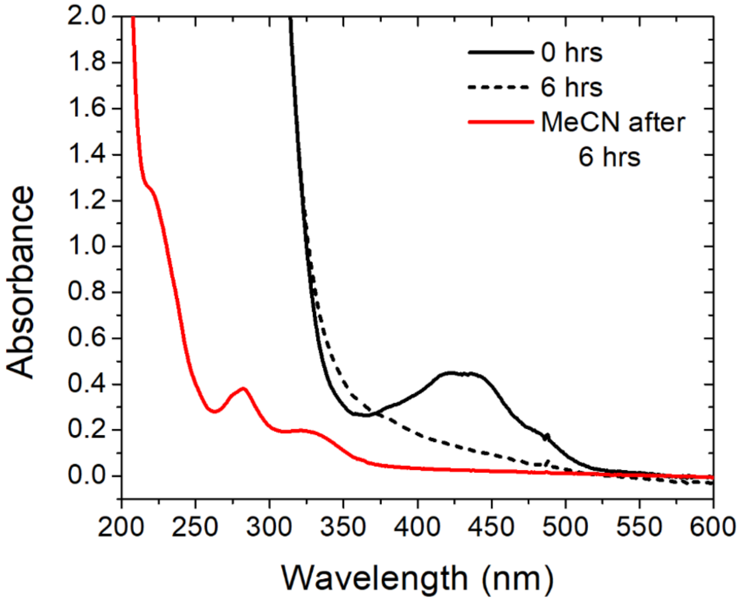

Figure S16. Absorbance spectra of 50% loaded ZrO_2-**A**-Zn with 25 cycles of TMA/H_2O before irradiation (black solid line) and after irradiation (black dashed line) as well as the absorbance spectrum of the MeCN solution after photolysis (red line).
